# Functional recovery continues beyond 3 months post‐basilar artery thrombectomy: A retrospective cohort study

**DOI:** 10.1111/cns.14182

**Published:** 2023-03-21

**Authors:** Lakshini Gunasekera, Peter Mitchell, Richard J. Dowling, Steven Bush, Bernard Yan

**Affiliations:** ^1^ Melbourne Brain Centre at Royal Melbourne Hospital University of Melbourne Parkville, Melbourne Victoria Australia; ^2^ Neurointervention Service, Department of Radiology Royal Melbourne Hospital Melbourne Victoria Australia

**Keywords:** basilar artery occlusion, modified Rankin score, stroke, thrombectomy

## Abstract

**Introduction:**

Untreated basilar artery occlusion (BAO) carries 70% mortality. Guidelines recommend thrombectomy with or without thrombolysis.

**Aim:**

We compared Modified Rankin Scores (mRS) at 3 and 12 months post thrombectomy to determine benefit of long‐term follow up.

**Methods:**

Retrospective, single centre analysis of BAO thrombectomies between 2015 and 2019. Inclusion criteria were symptomatic BAO on CT angiography, absent early ischemic changes, premorbid independence and intervention within 24 h. All received stroke ward care. Results were analysed with simple statistics and binary logistic regression as appropriate.

**Results:**

Of 82 patients: most were male (61%, 50/82) with median age 68 years (IQR 17 years) and median NIHSS 14 (IQR 15). Median door‐to‐puncture time was 42 min (IQR 72 min). Total deaths were 34.1% (28/82) at 3 months, and 37.8% (31/82) at 12 months. Of 51 patients alive at 12 months: 41% (21/51) had improved mRS, 16% (8/51) had worse mRS and 43% (22/51) had unchanged mRS, compared to 3 months. Improvements to mRS were: one point in 57.1% (14/21), two points in 28.9% (6/21) and three points in 4.8% (1/21). Nursing home admission was avoided in 11.8% (6/51) who improved from mRS4. Increased age was associated with decreased likelihood of reaching the primary outcome OR 0.87, 95% CI 0.76–0.99 (*p* value = 0.03).

**Conclusion:**

Over a quarter of patients improved beyond 3 months. Future studies should adopt long‐term follow up as primary outcome.

## Graphical abstract

Basilar artery occlusion is generally treated with mechanical thrombectomy; standard follow‐up occurs after 3 months. Longer term follow up at 12 months show that many patients continue to improve thus should not have their neurological fate sealed at 3 months. Future studies should adopt longer‐term 12 month outcomes as the primary end point.

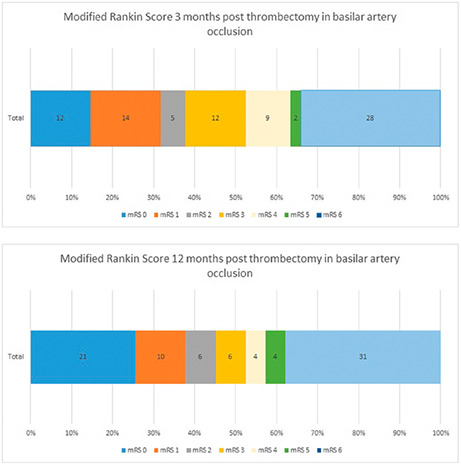

## 1 INTRODUCTION

Basilar artery occlusion (BAO) is a less frequent stroke subtype comprising roughly 1% of all strokes. It has significantly increased morbidity and mortality, approaching 70% if left untreated.^1^, ^2^, ^3^ Although mechanical thrombectomy improved the clinical outcomes of large vessel occlusions in the anterior circulation, there remained uncertainty about its efficacy in BAO.^1^ Two randomized controlled trials, Basilar Artery International Cooperation Study (BASIC) and Acute Basilar Artery Occlusion: Endovascular Interventions versus Standard Medical Treatment (BEST), investigated clinical outcomes between medical management versus mechanical thrombectomy but did not demonstrate the superiority of the latter.^4^, ^5^ However, the more recently published BASIC study has shown that 44% of patients treated with thrombectomy, compared to just 37% of medically managed patients, had favorable functional outcomes of modified Rankin score (mRS) 0–3 at 3 months.^6^ While there was no statistically significant difference between the groups, there is a trend toward favorable outcomes in mechanical thrombectomy.

On the other hand, Australian Urokinase Stroke Trial (AUST) randomized patients to anticoagulation versus intra‐arterial thrombolysis and showed a trend towards better outcomes in the endovascular treatment group.^7^ In addition, some studies have shown that a proportion of patients presenting with BAO demonstrated advanced age, low Glasgow coma scale, and high National Institute of Stroke Scale (NIHSS) score and still achieved functional independence post‐thrombectomy.^8^, ^9^, ^10^, ^11^ On this basis, endovascular treatment (either by mechanical thrombectomy or intra‐arterial thrombolysis) was included in 2019 acute stroke guidelines with level A recommendation.^2^

Studies show that BAO patients receiving thrombolysis continue to improve after the initial 3‐month monitoring period, for up to 2.8 years.^12^, ^13^ Similarly, thrombectomy leads to favorable outcomes at the 3‐month mark,^14^, ^15^, ^16^, ^17^ with one study even showing improvement at 12 months after ictal onset.^18^ Long‐term outcome data is useful in providing patients with hope, prognostication and further justifies the use of this procedure ‐ which incurs significant time and resources – given the absence of randomized trials in this area.

We performed a retrospective cohort analysis of BAO patients treated by thrombectomy to investigate the functional recovery comparing 3 and 12 months post‐index event.

## 2 METHODS

We performed a single‐center, retrospective analysis of patients with BAO who underwent thrombectomy during the study period of January 2015 to April 2019 at a comprehensive stroke center. The center is a public hospital with 24‐h access to mechanical thrombectomy.

Patient inclusion criteria as it related to thrombectomy eligibility included symptomatic BAO proven on computed tomography angiography, presentation within 24 h of ictal onset, absence of extensive early ischemic changes on noncontrast CT brain and favorable premorbid functional status. Exclusion criteria were non‐basilar artery strokes, presentation after 24 h after ictal onset, early ischemic changes on noncontrast CTB as this would lead to conservative management rather than thrombectomy, and unfavorable premorbid baseline leading to conservative management rather than endovascular therapy. Treatment proceeded at the neurointerventionalist's discretion with endovascular devices (Solitaire, Revive, Trevo, Penumbra, or Sophia aspiration catheters). Post‐procedure, all patients received subacute care on a specialist stroke ward with access to stroke nurses and daily allied health input.

**FIGURE 1:**
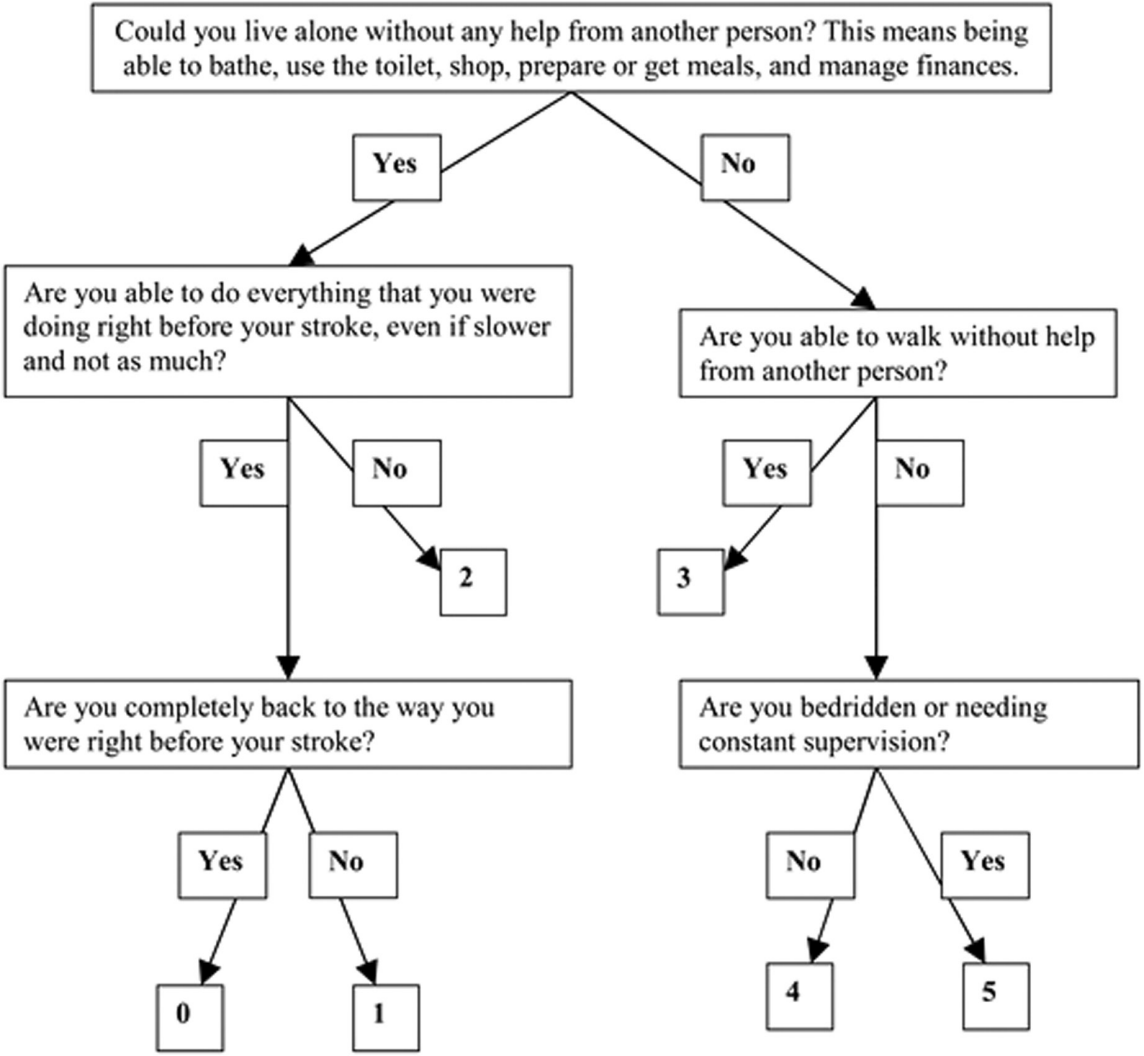
Revised version of simplified modified Rankin score questionnaire (Bruno et al.^19^).

For all patients included in the study, baseline, 3‐ and 12‐month mRS were collected. Baseline mRS scores were extracted from the initial stroke admission notes; 3‐ and 12‐month mRS scores were extracted from outpatient stroke clinic letters as documented by the treating neurologist. Where these letters were unavailable on our internal hospital network, they were requested by written correspondence from external hospitals where longer‐term patient data was available. If no formal mRS score was documented, two mRS‐certified‐assessors (L.G. and B.Y.) used the documented information to classify the most appropriate mRS using the algorithm in Figure 1, that has been validated by Bruno et al.^19^ Where no hospital or clinic follow‐up letters were available, patients were contacted directly on their listed home telephone number to perform a verbal telephone mRS which had previously been validated.^20^ We excluded patients with insufficient documentation to reliably ascribe an mRS score, where contact details were inaccurate and could not be retrieved, if they had since moved overseas or interstate with no new contact information, or did not have general practitioner or next‐of‐kin documented to derive a verbal mRS. Results have been reported as percentages using simple statistics and, where appropriate, binary logistic regression was completed with SPSS Statistics 22 (IBM). The level of statistical significance was considered at two‐tailed *p* < 0.05. We excluded patients with missing essential data from our analysis, so we did not apply imputation procedures for missing data.

## 3 RESULTS

### 3.1 Baseline demographics

There were 90 patients in our hospital mechanical thrombectomy database who had a diagnosis of basilar artery occlusion treated with thrombectomy during the study period. Of these 90 patients, we were able to obtain mRS at 3 and 12 months for 82 patients. The remaining eight patients were excluded due to the following reasons: 2/8 had moved overseas with no contact details available, 2/8 had moved interstate with no contact details available and 4/8 had their listed phone numbers disconnected. These eight patients lacked next‐of‐kin, family, or general practitioner details to track down their progress. This led to a final sample size of 82 patients in our final study. Please see Table 1 for baseline characteristics of the included patients. Most were male with median age of 68 years and presenting median National Institutes of Health Stroke Score 14. Most patients had intervention within an hour of presenting with median time from door to puncture of 42 min (IQR 72 min).

**TABLE 1:** Baseline characteristics of the study population.

	Frequency
Male sex	50/82 (61.0%)
Median age (IQR)	68 years (Q1 = 59 years, Q3 = 76 years, IQR = 17 years)
Comorbidities	
Hypertension	54/82, 65.9%
Hyperlipidemia	29/82, 35.4%
Atrial fibrillation	22/82, 26.8%
Diabetes	18/82, 22.0%
Ischemic heart disease	16/82, 19.5%
Median NIHSS (IQR)	14 (IQR 15)
Median door‐to‐puncture time (IQR)	42 min (72 min)

The pre‐morbid mRS scores were: mRS 0 (85.4%, 70/82), mRS 1 (8.5%, 7/82), mRS 2 (3.7%, 3/82), and mRS 3 (2.4%, 2/82).

### 3.2 Changes to modified Rankin scores over 12 months

A total of 34.1% (28/82) died within 3 months and 37.8% (31/82) total had died within 12 months of their stroke i.e. further three patients had died after 3 months. This left 62.2% (51/82) patients alive at 12 months post‐index event.

Of the patients who were alive after 12 months from index event: a total of 41% (21/51) had improved mRS at 12 months compared to 3 months; 16% (8/51) of patients had a worse mRS at 12 months than at 3 months and 43% (22/51) patients had an unchanged mRS score at these two time points.

When looking at the 21 patients with an improved mRS at 12 months: 57.1% (14/21) were better by one mRS point; 28.9% (6/21) were better by two mRS points and 4.8% (1/21) were better by three mRS points. No patients had improved by four or more mRS points. In total 11.8% (6/51) of patients alive at 3 months improved from mRS of 4 on longer‐term follow‐up which means they avoided nursing home admission.

Of the eight patients who were worse at the end of their 12‐month review compared to their 3‐month review, 62.5% (5/8) were worse by one mRS point, 25% (2/8) were worse by two mRS points and 12.5% (1/8) was worse by three mRS points.

**FIGURE 2:**
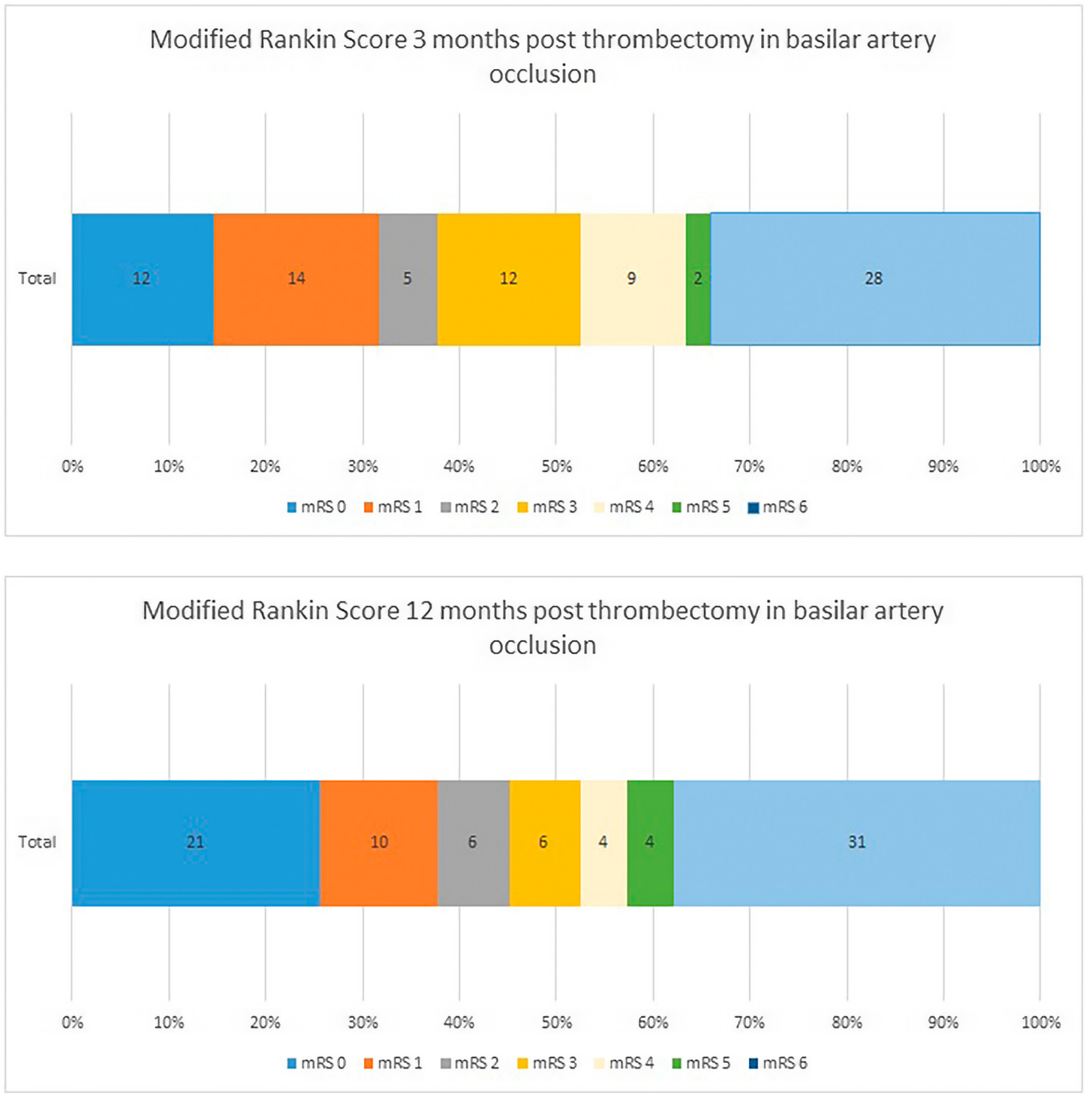
Comparison of modified Rankin scores at 3 versus 12 months post‐basilar artery thrombectomy.

See Figure 2 for visual representation of the changed mRS scores at 3 and 12 months.

Of 82 total patients included in our study, only 51 patients survived the initial 3 months. Of those who survived, 18 patients did not have initial NIHSS documented due to urgent intubation on arrival precluding full neurological assessment; another two patients did not have their initial NIHSS documented in the notes. In total, 31 patients who survived the initial 3 months had an admission NIHSS documented. In total 23/51 of patients surviving more than 3 months had an NIHSS <17, while eight patients had NIHSS >17. Of those with NIHSS >17, when comparing the 3‐month mRS to the 12‐month mRS, 3/8 (37.5%) had a worse mRS while 5/8 (62.5%) had stable mRS. There were no patients with NIHSS >17 who had a better mRS at 12 months compared to 3 months. Of the 23 patients with initial NIHSS <17, 12/23 (52%) had better mRS at 12 months compared to 3 months, 4/23 (17%) had worse mRS at 12 months and 7/23 (30%) showed stable mRS. This shows us that those with relatively minor stroke with NIHSS <17, are more likely to continue to improve after the initial 3 months when compared to someone with a larger stroke with NIHSS >17. This can be used to inform patients of their likely prognosis at the time of clinical reviews.

### 3.3 Statistical analysis

Binary logistic regression was used to test the impact of age and initial NIHSS on the primary outcome (proportions reaching mRS 0–2 within 12 months). Increasing age was associated with decreased likelihood of reaching the primary outcome OR 0.87, 95% CI 0.76–0.99 (*p* value = 0.03). The association between NIHSS and the primary outcome did not reach statistical significance.

Sensitivity analysis was conducted to ascertain if age <50 years was positively associated with the primary outcome. While our sample size limited analysis to only four patients <50 years, all were able to achieve functional independence with mRS 0–2 at 12 months. Further studies with larger sample sizes would be required to ensure validity of these results.

### 3.4 Strengths of the study and its implications

This was a real‐world analysis of long‐term outcomes after mechanical thrombectomy in basilar artery occlusion. There are limited studies looking at long‐term outcomes for this rare stroke subtype.^18^ Our study adds to this body of literature and strengthens the perception that this patient population does not have their neurological fate sealed at 3 months as previously thought.^21^

Our study has demonstrated that such patients may go on to achieve better functional outcomes than their 3‐month mRS score would suggest. This included a quarter of our total patients (25.6%, 21/82) or nearly half of the patients who were alive after 1 year (41.2%, 21/51). This is useful in delivering information to patients and families about the likely trajectory after this diagnosis – we must not lose hope after the first 3 months.

Less than half (43.1%, 22/51) recorded the same mRS after 12 months at their initial review. This suggests that the mRS at 3 months should not lead to cessation of active supports because good outcomes remain possible. While the difference between mRS 1 and mRS 0 may be modest, we cannot discount the positive impact of recovery to baseline neurology for each individual person. Furthermore, the 11.8% (6/51) who improved from mRS 4 show that in over 10% of those alive at 3 months, it is possible to improve to such an extent that nursing home admission can be avoided.

In addition, recent studies in BAO treated by endovascular thrombectomy showed that nearly 80% had favorable functional outcomes at 6 months,^22^, ^23^ and even 1 year from the index stroke.^24^ These findings were buttressed by trials of intracerebral hemorrhage, Early surgery versus initial conservative treatment in patients with spontaneous supratentorial lobar intracerebral hematomas (STICH II) and efficacy and safety of minimally invasive surgery with thrombolysis in intracerebral hemorrhage evacuation (MISTIE), which showed favorable outcomes 12 months after initial hemorrhage, and that recovery did not plateau at the 3‐month time point.^25^, ^26^ These findings suggested that 3‐month Modified Rankin Score (mRS) may be premature and that withholding rehabilitation may result in opportunities lost in functional recovery. Furthermore, patients with locked‐in‐syndrome deem their life to have higher quality than that judged by doctors,^27^, ^28^ which indicates a need for health professionals to lead with more hope and active management strategies, rather than palliative approaches.

The strengths of this study are that it fills a gap in the literature where there is paucity of evidence about long‐term functional outcomes in basilar artery occlusion patients treated with thrombectomy. It has a large sample of 82 patients presenting with a rare subtype treated with a thrombectomy procedure only available at a comprehensive stroke center.

The results suggest that this patient subset should have their mRS measured at 12 months rather than 3 months as the latter is not an accurate representation of long‐term prognosis. Other strengths include that this is a real‐life sample of post‐thrombectomy patients at a tertiary hospital in which thrombectomy is available 24 h per day by experienced neuro‐interventionalists. We have obtained results that are likely to inform future practice in advising patients about long‐term outcomes in this stroke subtype and also engage more heavily in rehabilitation for patients for a year after their initial treatment due to ongoing gains seen. Our inclusion of all patients who received thrombectomy for this stroke subtype, as long as their 12‐month outcomes were known, aimed to avoid selection bias.

### 3.5 Limitations of study and future directions

Limitations include retrospective nature of study and single site which may preclude application of results to other populations.

The results of this study provide confidence in thrombectomy for basilar artery occlusion as a proportion will further improve to a state of independence as indicated by mRS at 12 months. The reasons for ongoing improvement may be attributable to the long‐term benefits of rehabilitation. However, future studies would be required to validate and to clarify which subgroup of patients would best benefit from rehabilitation. Other thrombectomy studies have looked into other factors such as renal function,^29^ the use of thrombectomy with or without alteplase,^30^ and even the local ischemic milieu that may affect long‐term outcomes.^31^, ^32^ We hope that our study will add to this body of literature to ascertain the optimal factors to achieve good long‐term functional outcomes for patients.

## 4 CONCLUSION

We have shown that a significant proportion of patients with BAO treated with thrombectomy improve after their initial 3‐month mRS review. We suggest a 12‐month follow‐up mRS as primary outcome in future BAO studies. Such patients should also be advised that they can continue to improve despite a modest mRS at 3 months.

## AUTHOR CONTRIBUTIONS

BY and LG conceived the study. LG performed data collection. LG analyzed data. LG drafted the manuscript and all authors contributed equally to its revision. BY and LG take responsibility for the paper as a whole.

## CONFLICT OF INTEREST STATEMENT

None declared.

## Data Availability

Data available at reasonable request.
